# Topographical Disorientation: Clinical and Theoretical Significance of Long-Lasting Improvements Following Imagery-Based Training

**DOI:** 10.3389/fnhum.2019.00322

**Published:** 2019-09-20

**Authors:** Maddalena Boccia, Alessia Bonavita, Sofia Diana, Antonella Di Vita, Maria Paola Ciurli, Cecilia Guariglia

**Affiliations:** ^1^Cognitive and Motor Rehabilitation and Neuroimaging Unit, IRCCS Fondazione Santa Lucia, Rome, Italy; ^2^Department of Psychology, “Sapienza” University of Rome, Rome, Italy; ^3^Department of Human Neuroscience, “Sapienza” University of Rome, Rome, Italy; ^4^Neuropsychology Unit, IRCCS Fondazione Santa Lucia, Rome, Italy

**Keywords:** acquired topographical disorientation, brain damage, spatial navigation, topographical memory, episodic memory

## Abstract

Neuropsychological studies on acquired topographical disorientation have provided useful insights into the contribution of different brain regions to human navigation. However, little is known about the possibility to restore navigational skills after brain damage. Here we describe the case of No Longer Lost (NLL), a 49-year-old man who complained of severe topographical disorientation following traumatic brain injury. Extensive neuropsychological evaluation at baseline revealed selective episodic memory deficits and topographical disorientation. NLL underwent 8-week imagery-based treatment (IBT) inspired by current cognitive models of human spatial navigation. After IBT, NLL improved topographical skills and episodic memory. From a clinical point of view, the present study describes a model-based intervention for topographical disorientation. From a theoretical point of view, it provides new insights into the cognitive models of human spatial navigation and straightforward evidence about common phylogenetic roots of brain mechanisms devoted to spatial navigation and memory.

## Background

To orient themselves within the environmental space – namely, the space beyond the sensory horizon (Wolbers and Wiener, 2014) – individuals have to process “online” information about their own position and facing direction (Sulpizio et al., 2017, 2018), as well as to recall previously acquired “offline” information about the environment (Wolbers and Hegarty, 2010). Physiological evidence suggests that “online” information about position and facing direction is coded by distinct neural populations, namely by hippocampal place cells and head-direction cells, respectively (O’Keefe and Dostrovsky, 1971; Taube, 1998; Ekstrom et al., 2003; Vass and Epstein, 2013; Sulpizio et al., 2014). “Offline” environmental knowledge may be represented as Landmark, Route, and Survey representation (Siegel and White, 1975). Landmark representation roughly corresponds to the figurative memory of environmental objects, through which individuals “beacon” toward salient landmarks. Route representation concerns the memory of paths connecting landmarks and is organized according to an egocentric frame of reference. Survey representation implies the encoding of directions and distances between landmarks regardless of the individual’s position, resembling a map-like representation of the environment. Recent neuroimaging evidence supports this model (Boccia et al., 2016).

Following the BBB model (Byrne et al., 2007), neural populations within the posterior parietal lobe – namely, “egocentric parietal window” (p. 345) – maintain the head-centered egocentric map of the space and provide exclusive access into long-term navigational memory stored in the medial temporal lobe; in the medial temporal lobe, the parahippocampal gyrus develops allocentric (survey) representations and the hippocampus stores long-term spatial memories; finally, a transformation circuit in the retrosplenial cortex allows to transform the spatial representations stored in an allocentric format into an egocentric (route) format, and vice versa. Evidence in nonhuman primates (Kravitz et al., 2011) and humans (Boccia et al., 2014, 2017a), and neuropsychological studies (Aguirre and D’Esposito, 1999) support this model. Specifically, lesions of the posterior parietal cortex yield to egocentric disorientation, namely a deficit in representing the location of objects with respect to the self (Holmes and Horrax, 1919; Levine et al., 1985; Stark et al., 1996). Instead, lesions of the retrosplenial cortex lead to heading disorientation: patients lose the sense of direction and are unable to direct toward locations beyond the vista space (Takahashi et al., 1997), namely the space that can be explored at a glance (Montello, 1993; Wolbers and Wiener, 2014). Landmark recognition deficits are widely reported following lesions in the lingual gyrus: patients fail in recognizing and representing salient environmental stimuli, even in absence of perceptual deficits (Aguirre and D’Esposito, 1999). These neuropsychological findings provide important information about the unique and causal contribution of each specific node of the parieto-medial temporal lobe network of spatial navigation in humans. However, evidence for a redundant code within this network (Ekstrom et al., 2017; Boccia et al., 2019) suggests the possibility that, in presence of a lesion in a specific node, compensation mechanisms may allow the recovery of the impaired process.

Here we describe the case of NLL (acronym for No Longer Lost), a patient with acquired topographical disorientation, who underwent a novel imagery-based neuropsychological rehabilitation for spatial navigation, tailored on the patient’s neuropsychological profile and the theoretical framework described above.

## Case Presentation

NLL, a 49-year-old man working as a realtor, suffered from an extensive head trauma (including facial trauma) and coma, which lasted about 1 week, following a motor vehicle accident. NLL referred to the IRCCS Santa Lucia in Rome 4 years after his accident due to persistent topographical disorientation, even in highly familiar environments, and memory deficits. The CT performed immediately after the event, and the MRI performed 4 years later, revealed a lesion of the right temporal lobe extending also to subcortical areas (Supplementary Figure 1). No neuropsychological evaluation has been performed immediately after the accident or in the following 4 years.

NLL reported that topographical disorientation greatly impacted over his life and professional duties, and that soon after the event he realized he was unable to recognize landmarks and routes, and adopted several compensatory strategies (e.g., he wrote little numbers on the outside wall of the houses he had to visit with customers, he mentally counted the number of bus stops he had to pass to get to the correct place). Also, he reported to be unable to imagine landmarks and routes connecting them, in both familiar and novel environments. Furthermore, he reported episodic memory deficits: he was impaired in recalling events, including, for example, the customers he met or the houses he visited; he was unable to retrieve memories about previous travels reporting that “it is as if I’ve never been there … There is no trace about that in my memory.” He also reported to be unable to talk about relevant daily events, for example with his father, when he called NLL in the evening, because he could not recall them.

The study was designed in accordance with the principles of the Declaration of Helsinki and was approved by the ethical committee of the IRCCS Fondazione Santa Lucia. Informed consent was obtained from the patient.

### Neuropsychological Assessment at Baseline

#### General Neuropsychological Assessment

NLL performed well within the normal range in tests assessing attention, intelligence, executive functions, language, and working memory (Table 1). He was impaired in tests evaluating visuo-spatial learning and delayed recall (*Corsi Block Tapping Test*, CBT; Corsi, 1972; Table 2) and visual, spatial, and verbal memory in ecological contexts (RBMT-3; Wilson et al., 2008; Table 3).

**TABLE 1:** General neuropsychological assessment.

**Test**	**NLL’s score**	**ES^a^/PR^b^/SS^c^**	**Cut-off**
	**(baseline)**		
**Attention**			
**Trail making test**			
A	55 s	2^a^	
B	90 s	4^a^	
B−A	35 s	4^a^	
Stroop			
Errors	0	4^a^	
Time	14	4^a^	
TEA			
Alertness – tonic	298	10^b^	
Phasic	237	38^b^	
Median	0.239	99^b^	
Go-NoGo median	543	42^b^	
False alarms	0	>46^b^	
Divided attention median	740	12^b^	
Omissions	**5**	**4**^b^	
False alarms	**2**		
Working memory median	684	28^b^	
Omissions	3	<16^b^	
False alarms	0	>76^b^	
***PASAT***			
**Stimulus interval 3000**			
Correct answers	46		44.2
Errors	7		
Omissions	7		
Percentage of errors	23		
**Stimulus interval 2600**			
Correct answers	47		38.69
Errors	6		
Omissions	7		
Percentage of errors	22		
**Stimulus interval 2200**			
Correct answers	47		34.77
Errors	3		
Omissions	10		
Percentage of errors	22		
**Stimulus interval 1800**			
Correct answers	37		28.07
Errors	0		
Omissions	23		
Percentage of errors	38		
**Intelligence**			
Raven progressive matrices	36	4^a^	
**Executive functions**			
**Wisconsin card sorting test**			
Errors (%)	15	61^b^	
Perseverative errors (%)	8	63^b^	
Non-perseverative errors (%)	7	58^b^	
Categories completed	6	>16^b^	
Number of attempts on the first category	12	>16^b^	
Inability to maintain the set	0	>16^b^	
Learning to learn	+	>16^b^	
**Tower of London**			
Correct responses (total)	7	118^c^	
Moves (total)	8	120^c^	
Planning time (total)	88	114^c^	
Execution time (total)	175	106^c^	
Total time	263	100^c^	
Time-limit breaks	0	104^c^	
Rule breaks	0	108^c^	
**Language**			
**Fluency**			
Phonemic	38	4^a^	
Semantic	61	4^a^	
Alternate	46	4^a^	
Shifting	0.93	4^a^	
**Memory**			
Digit span (forward)	7	4^a^	
**Rey auditory verbal learning test**			
Immediate recall	44	3^a^	
Delayed recall	8	2^a^	
Recognition	12		
**Rey–Osterrieth figure**			
Copy	36	4^a^	
Immediate recall	20	3^a^	
Delayed recall	21	3^a^	
Oblivion	−1	4^a^	
**SMIRNI**			
Words	24	10–25^b^	
Buildings	25	25–50^b^	
Faces	25	25–50^b^	
**Space and object perception**			
***VOSP***			
Screening	20		15
Incomplete letters	20		17
Silhouettes	26		16
Objects decision	19		15
Progressive silhouettes	**13**		**14**
Dot counting	10		8
Position discrimination	20		8
Number of location	10		7
Cube analysis	10		6

*Tests are standardized for use with Italian-speaking individuals. Pathological scores are marked in bold. ^*a*^Equivalent scores adjusted for sex, age, and years of education. ^*b*^Percentile rank. Performances below the 5th percentile and equivalent score equal to zero should be considered pathological. ^*c*^Standard scores; performances below 84 should be considered pathological.*

**TABLE 2:** Performances on DDTDB.

	**Baseline**		**Post-treatment**		**Follow-up**	
	**NLL’s score**	**Crawford analysis**	**NLL’s score**	**Crawford analysis**	**NLL’s score**	**Crawford analysis**
**Corsi Block Tapping Test (CBT)**						
Span	5^a^	*t* = 0.047, *p* = 0.481^∗^				
Learning	46^a^	*t* = −3.187, *p* = 0.001^∗^	134^a^	*t* = 1.065, *p* = 0.146^∗^	127^a^	*t* = 0.727, *p* = 0.235^∗^
Delayed recall	3^a^	*t* = −1.681, *p* = 0.050^∗^	6^a^	*t* = −0.280, *p* = 0.390^∗^	8^a^	*t* = 0.654, *p* = 0.258^∗^
**Walking Corsi test (WalCT)**						
Span	5^a^	*t* = 0.000, *p* = 0.500^∗^				
Learning	50^a^	*t* = −3.995, *p* < 0.001^∗^	128^a^	*t* = 0.582, *p* = 0.282^∗^	101^a^	*t* = −1.002, *p* = 0.161^∗^
Delayed recall	8^a^	*t* = 0.412, *p* = 0.341^∗^	6^a^	*t* = −1.650, *p* = 0.053^∗^	8^a^	*t* = 0.412, *p* = 0.341^∗^
**Cognitive map test (CMT)**						
Learning (CMT-L)	1500s^b^	*z* = 2.686, *p* = 0.047^#^	720s^k^	*z* = −0.806, *p* = 0.461^#^	1380s^b^	*z* = 2.188, *p* = 0.091^#^
Recall (CMT-R)	17.44s^c^	*z* = −1.706, *p* = 0.139^#^	38.72s^i^	*z* = −1.340, *p* = 0.436^#^	19.27s^c^	*z* = −1.563, *p* = 0.169^#^
Road map test	28^d^	*t* = −0.345, *p* = 0.372^∗^				
**3D mental rotation**						
Accuracy	38^e^	*z* = 1.046, *p* = 0.251^#^				
Response time	6955.33ms^f^	*z* = 1.641, *p* = 0.148^#^				
**Navigational tasks in real environment**						
Landmark recognition	15^g^	*z* = 0.202, *p* = 0.461^#^				
Route strategy	7^h^					
Throwback	6/6^i^					
Map-following task	3/4^j^	*z* = −0.827, *p* = 0.342^#^	4/4^j^		4/4^j^	

*For each task and assessment (baseline, post-treatment, and follow-up) NLL’s raw score is reported, along with the result of the Crawford analysis [^∗^Crawford and Garthwaite (2002); ^#^Crawford et al. (2011)]. ^*a*^Normative data from Piccardi et al. (2013). ^*b*^Normative data from 29 males (*M* = 542.18, *SD* = 275.91). ^*c*^Normative data from 29 males (*M* = 20.76s, *SD* = 8.49). ^*d*^Normative data from six males (*M* = 29.00, *SD* = 2.68). ^*e*^Normative data from 29 males (*M* = 34.74, *SD* = 7.03). ^*f*^Normative data from 29 males (*M* = 8935.90s, *SD* = 355.26). ^*g*^Normative data from 13 males (*M* = 12.77, *SD* = 2.62). ^*h*^Normative data from 13 males (*M* = 6.08, *SD* = 0.95). ^*i*^Normative data from five males (*M* = 5.80, *SD* = 0.45). ^*j*^Normative data from 13 males (*M* = 3.77, *SD* = 0.60). ^*k*^Normative data from six males (*M* = 580s, *SD* = 256.43). ^*l*^Normative data from six males (*M* = 21.19s, *SD* = 8.66).*

**TABLE 3:** Rivermead Behavioral Memory Test-3.

**Subtest**	**NLL’s score**	**NLL’s score**
	**(baseline)**	**(post-treatment)**
Names	6	7
Belongings	6	8
Appointments	4	4
Picture recognition	14	14
Story recall – immediate	**5**.**5**	9
Story recall – delayed	**4**.**5**	8.5
Face recognition – delayed	14	10
Route recall – immediate	13	13
Route recall – delayed	**8**	13
Messages – immediate	6	6
Messages – delayed	6	6
Orientation and date	14	13
Novel task – immediate	**18**	48
Novel task – delayed	**6**	17
GMI	83	104

*Pathological scores are marked in bold. GMI, general memory index.*

### Assessment of Navigational Abilities

Navigational abilities were tested using the *DiViNa Developmental Topographical Disorientation Battery* (DDTDB), which is extensively described in previous works (Bianchini et al., 2010, 2014; Palermo et al., 2014) and is based on theoretical models of human navigation (Siegel and White, 1975; Wang and Spelke, 2002). NLL’s scores were compared with those of men in our database or previous published studies (Table 2), by using *t*-test modified procedure (Singlims.exe; Crawford and Garthwaite, 2002). Age and education were included as covariates (BTD_Cov.exe; Crawford et al., 2011) when necessary.

#### Walking Corsi Test (WalCT; Piccardi et al., 2008, 2013)

Both short-term and long-term memory in navigational vista space (i.e., learning and delayed recall) were assessed. NLL performed as well as controls in short-term memory and delayed recall. However, he was impaired in learning spatial positions (Table 2).

#### Cognitive Map Test (CMT; Iaria et al., 2007)

We assessed both the formation/learning (CMT-L) and the recall (CMT-R) of a cognitive map, asking NLL to travel in a virtual city, in which there were six landmarks (i.e., a cinema, a restaurant, a bar, a hotel, a pharmacy, and a flower shop) (for full description of the task and virtual environment, see Iaria et al., 2007). NLL performed worse than controls on CMT-L, but comparably to controls on CMT-R (Table 2).

#### Route Strategy and Landmark Recognition

Experimenter shows a path in real environmental space, asking the participant to pay attention to all environmental objects. Immediately after, the participant is blindfolded and brought back to the starting point; then she/he is asked to discriminate among distractors (*N* = 8) the specific landmarks (*N* = 8) encountered along the path (maximum score = 16). Finally, the participant has to reproduce the path shown by the examiner (for similar procedure, see Conson et al., 2018). The score is calculated by summing the segments correctly retraced (maximum score = 7). NLL correctly performed landmark recognition (Table 2) and retraced the route without errors (Table 2), even though he claimed he had no clear navigational goal and was unable to imagine the successive steps of the path.

#### Throwback to the Starting Point

Participant is asked to go back to the starting point of a route previously shown by the examiner. The score corresponds to the number of segments correctly performed (maximum score = 6). NLL’s performance was errorless.

#### Map-Following Task

The participant is provided with a map of a real environmental navigational space on which a starting point and a goal are depicted, and asked to draw the shorter path to reach the goal. Then the participant is brought to the starting point and asked to use the map to reach the goal, following the path he/she drew. The score corresponds to the number of segments correctly performed following the path participant drew (maximum score = 4). NLL hesitated at crossroads, but his performance (3/4 correct segments) did not differ from that of controls (Table 2), and he correctly recognized his errors and difficulties.

#### Mental Imagery Skills

NLL complained of deficits in imaging landmarks and routes; thus, we tested his mental imagery skills by using the *Complete Visual Mental Imagery Battery* (CVMIB) (Palermo et al., 2016), which allows to assess systematically the ability to generate, maintain, inspect, and transform visual mental images (for a complete description of tests and normative data, see Palermo et al., 2016). For each subtest (Buildings, Objects, Color 1, Color 2, and Color 3, Inspection of Objects and Letters, Folding, and the Mental Rotation), the sum of the correct answers was computed (Table 4). NLL performed worse than controls on Object and Color 2 subtests (Table 4); he performed within the normal range in the remaining subtests (Table 4). Also, Road Map Test (*RMT*; Money et al., 1965) and *3D Mental Rotation Task* (*MRT*; Thurstone, 1937) were performed comparably to controls.

**TABLE 4:** Complete visual mental imagery battery at baseline.

**Process**	**Subtest**	**Maximum**	**Control**		**NLL’s score**	**Crawford analyses**	
		**score**	**(Mean and SD)^a^**		**(baseline)**	**(*t* and *p*)**	
Generation	Buildings	20	19.28	1.04	18	–1.209	0.119
	Objects	20	19.60	0.78	18	–2.016	0.027
Maintenance	Color 1	10	9.57	0.57	10	0.741	0.233
	Color 2	10	9.82	0.39	9	–2.066	0.024
	Color 3	10	9.57	0.74	9	–0.757	0.228
Inspection	Objects	20	18.67	1.18	18	–0.558	0.291
	Letters	20	19.50	1.10	20	0.447	0.329
Transformation	Folding	20	16.75	2.93	12	–1.593	0.062
	Mental rotation	20	16.71	2.66	14	–1.001	0.163

*^*a*^Normative data are derived from Palermo et al. (2016).*

### Intervention Hypothesis

The neuropsychological assessment revealed a specific deficit in the formation of the cognitive map. Cognitive maps are pivotal for spatial orientation, since they allow individuals to reach any destination within the environmental space. Individuals unable to form a cognitive map get lost more frequently than individuals who are able to form and use cognitive maps of the environment (Iaria and Barton, 2010). Based on NLL’s complaints, as well as on evidence on the pivotal role of Mental Imagery in environmental navigation, we submitted him to an imagery-based treatment (IBT), tailored on NLL’s difficulties and aimed at improving and restoring navigational skills, with possible generalization to the episodic memory domain. The IBT was inspired by the imagery-based intervention proposed by Kaschel et al. (2002) for memory rehabilitation. Evidence from current cognitive models of spatial navigation and psychophysiological findings were integrated in the IBT (Siegel and White, 1975; Wang and Spelke, 2002; Wolbers and Wiener, 2014). Specifically, IBT followed the developmental hierarchical model proposed by Siegel and White (1975), neurophysiological evidence about head-direction and place coding (O’Keefe and Dostrovsky, 1971; Taube, 1998) and evidence about the impact of the spatial scale (Wolbers and Wiener, 2014). Thus, we developed training activities allowing to progressively move from the figurative memory of landmark representation in the vista space, to the egocentric route-based and allocentric survey-based representation of the environmental space, retracing ontogenetic acquisition stages proposed by Siegel and White (1975). IBT is extensively described in the Supplementary Material. In brief, in a first phase NLL was motivated to the imagery training and acquired the ability to rapidly generate mental images. Then, he underwent a second phase during which he was asked to generate and retrieve navigational mental images of landmarks, routes, and environmental map-like representations (i.e., survey representations).

### Post-treatment Neuropsychological Assessment

After 8 weeks of treatment, the tests in which NLL performed worse than controls were repeated. For all navigational, spatial, and memory tests, alternate versions were used. The only test for which alternate versions were not available is the CVMIB. However, no feedback is provided to participants during the execution of the test and possible test/re-test effects are unlikely. Effectiveness, namely the measure reflecting the potential improvement achievable during rehabilitation, was calculated for each test, as it follows:

$$\mathrm{Effectiveness}=\frac{\mathrm{Post}\,\mathrm{treament}\,\mathrm{score}-\mathrm{Baseline}\,\mathrm{score}}{\mathrm{Maximum}\,\mathrm{score}-\mathrm{Baseline}\,\mathrm{score}}*100$$

Learning of spatial positions within reaching and navigational vista spaces significantly improved, with an effectiveness of 89.79 and 82.97% on the CBT and the WalCT, respectively; after IBT performances on both tests were comparable with those of the control group (Table 2). Also, after IBT NLL did not differ from the control group on an alternate version of the CMT (Table 2). His performance on an alternate version of the map-following task was errorless (four out of four segments correctly executed), with an effectiveness of 100%. Improvement was stable 8 months later (Table 2).

Interestingly, after IBT NLL performed within the normal range on ecological memory tests (RBMT-3) in which he initially performed below the cut-offs (effectiveness on General Memory Index = 32.81%), suggesting that improvement on navigational skills also generalized to episodic memory (Table 3).

Immediately after the IBT, NLL also performed at ceiling on the Object Generation and Color subtests of the CVMIB, with an effectiveness of 100% in both subtests.

## Discussion

When NLL came to our observation he was seriously worried about his topographical disorientation and memory impairment. At baseline, he showed deficits in topographical and visuo-spatial learning and an inability to form a cognitive map of environmental space. We also found a specific deficit in generating mental images of objects and in maintaining them. Performances on landmark recognition, route learning, and map-following tasks were well within the normal range, even if they were performed with evident efforts. Neuropsychological evaluation highlighted a deficit in learning and retrieving structured verbal material (i.e., stories), despite an intact ability to learn and recall unstructured verbal material, (i.e., word lists). IBT yielded a significant improvement in all the areas in which NLL was impaired. Indeed, after 8 weeks of IBT, NLL’s performances did not differ from controls’ ones in visuo-spatial and topographical learning, suggesting that IBT fostered memory for positions within both reaching and vista space; he also performed without differences from controls on CMT, suggesting that NLL recovered the ability to form a cognitive map of the environmental space. Accordingly, after IBT he ameliorated his ability to use a map for navigating the environmental space, performing at ceiling on the map-following task. Interestingly, also episodic memory improved over the original performance: after IBT NLL’s performances on learning and recalling a story fell well within the normal range. These results deserve great attention in the light of possible clinical applications to topographical disorientation and episodic memory deficit as well as for their theoretical implications.

Despite the single case methodology, present findings provide a unique Contribution To The Field of neuropsychological rehabilitation of topographical disorientation. To the best of our knowledge, there is no evidence about effective neuropsychological rehabilitation of topographical disorientation. The only papers describing the rehabilitation of this disorder, indeed, focused on the acquisition of compensatory strategies (Incoccia et al., 2009; Bouwmeester et al., 2015; Svoboda et al., 2018); in some cases (Svoboda et al., 2018), the intervention was just aimed to allow patients to autonomously navigate in very familiar environments; in others, no generalization occurred for untreated materials (Davis and Coltheart, 1999). However, topographical disorientation may have a great impact on individual functioning. As it happened for NLL, people with topographical disorientation experience great difficulties which may prevent full recovery of autonomies. Before IBT, NLL used compensatory non-spatial strategies to cope with his daily life activities and professional duties, which likely resulted in non-pathological performances on ecological assessment of navigational skills (i.e., performance on landmark recognition, route learning, and map-following tasks). However, the compensation did not fully prevent NLL from getting lost and experiencing daily life difficulties. Indeed, he failed in processing and acquiring proper spatial information, such as positions, as also demonstrated by pathological scores on WalCT. After IBT, NLL was able to learn and recall spatial positions within the environmental, vista, and reaching spaces, suggesting that the neuropsychological rehabilitation protocol we developed was able to restore the spatial mechanisms disrupted by traumatic brain injury.

Spatial information is processed by a redundant code in the brain and the interaction between different areas is crucial for successfully navigating within the environmental space (Ekstrom et al., 2017). IBT likely taps on the wide network of areas involved in generating mental images of environmental space, including the hippocampus, the retrosplenial cortex and the parahippocampal place area (Boccia et al., 2015, 2017b). Considering that spatial navigation and mental imagery of familiar places arise from the interaction between these brain areas (Boccia et al., 2016, 2017b, 2019) it is possible that IBT, fostering the interaction between these regions, allowed to restore mechanisms of environmental navigation on account of the redundant code within this network (Boccia et al., 2019). This interpretation is consistent with lesion location and extension of NLL, which mainly involved the right temporal lobe, sparing other nodes of the parieto-temporo medial network of spatial navigation (Boccia et al., 2017a).

Besides the severe topographical disorientation, NLL was also affected by a severe episodic memory deficit. Interestingly, the effect of IBT generalized to episodic memory. Previous evidence supports the use of imagery-based trainings for neuropsychological rehabilitation of memory (Piras et al., 2011). Most randomized control trials (RCTs) used visual imagery and visualization of stories (Chiaravalloti et al., 2005) of relevant everyday materials (Kaschel et al., 2002). Here we found that imagery-based training of spatial abilities may improve performance on the recall of a story. Also, NLL reported that recall of relevant everyday events was significantly improved after IBT. He said “… now I’m able to tell my father what happened during the day when he calls me in the evening.” This result ties well with the recent hypothesis that mechanisms of planning and memory have their phylogenetic roots within mechanisms of spatial navigation in the physical world (Buzsaki and Moser, 2013). According to this view episodic memory has evolved from mechanisms of environmental navigation, and shares the same neuronal algorithms used to navigate within the real environment. In this vein, the improvement we found for episodic memory may be due to the restoring of imbalanced spatial “root” mechanisms. Also, this result is consistent with the idea that “spatial navigation serves as a model system to identify key coding principles governing cognitive spaces” (Bellmund et al., 2018, p. 362).

An alternative explanation for NLL’s disabilities is related to his visual imagery deficits: a lesion in the right temporal region may cause the slight deficit we observed at baseline in generating and maintaining mental images; accordingly, NLL complained of a deficit in imaging landmarks and routes. This deficit might have caused a mixed spatial and episodic memory deficit. In this light, the recovery of spatial and episodic memory deficits may be mediated by the recovery of mental imagery skills we detected at the post-treatment evaluation. However, it has to be noted that we observed only a slight imagery deficit at baseline, in contrast to NLL’s dramatic disability in navigating within highly familiar and novel environments and in remembering his own life events.

Even if compelling, present results deserve caution, since the effects we detected are based on the observation of a single case. Thus, further systematic investigations of imagery-based rehabilitation protocols for spatial navigation and memory (especially by means of RCTs) are needed to draw definite conclusions.

## Data Availability

The datasets generated for this study are available on request to the corresponding author.

## Ethics Statement

The studies involving human participants were reviewed and approved by Local ethical committee of the IRCCS Fondazione Santa Lucia in Rome. The patients/participants provided their written informed consent to participate in this study. Written informed consent was obtained from the individual(s) for the publication of any potentially identifiable images or data included in this article.

## Author Contributions

All the authors significantly contributed to the work, read, and approved the final version of the manuscript. AD and MPC performed the neuropsychological assessment. MB, SD, AD, and CG conceived the IBT. SD and AB conducted the IBT. MB and AB ran the statistical analyses and wrote the first draft of the manuscript.

## Conflict of Interest Statement

The authors declare that the research was conducted in the absence of any commercial or financial relationships that could be construed as a potential conflict of interest.

## References

[B1] Aguirre G. K. D’Esposito M. D. (1999). Topographical disorientation: a synthesis and taxonomy. *Brain* 122 1613–1628. 10.1093/brain/122.9.1613 10468502

[B2] Bellmund J. L. S. Gardenfors P. Moser E. I. Doeller C. F. (2018). Navigating cognition: spatial codes for human thinking. *Science* 362:eaat6766. 10.1126/science.aat6766 30409861

[B3] Bianchini F. Incoccia C. Palermo L. Piccardi L. Zompanti L. Sabatini U. (2010). Developmental topographical disorientation in a healthy subject. *Neuropsychologia* 48 1563–1573. 10.1016/j.neuropsychologia.2010.01.025 20144632

[B4] Bianchini F. Palermo L. Piccardi L. Incoccia C. Nemmi F. Sabatini U. (2014). Where Am I? A new case of developmental topographical disorientation. *J. Neuropsychol.* 8 107–124. 10.1111/jnp.12007 23336564

[B5] Boccia M. Guariglia C. Sabatini U. Nemmi F. (2016). Navigating toward a novel environment from a route or survey perspective: neural correlates and context-dependent connectivity. *Brain Struct. Funct.* 221 2005–2021. 10.1007/s00429-015-1021-z 25739692

[B6] Boccia M. Nemmi F. Guariglia C. (2014). Neuropsychology of environmental navigation in humans: review and meta-analysis of fMRI studies in healthy participants. *Neuropsychol. Rev.* 24 236–251. 10.1007/s11065-014-9247-8 24488500 PMC4010721

[B7] Boccia M. Piccardi L. Palermo L. Nemmi F. Sulpizio V. Galati G. (2015). A penny for your thoughts! patterns of fMRI activity reveal the content and the spatial topography of visual mental images. *Hum. Brain Mapp.* 36 945–958. 10.1002/hbm.22678 25359694 PMC6869538

[B8] Boccia M. Sulpizio V. Nemmi F. Guariglia C. Galati G. (2017a). Direct and indirect parieto-medial temporal pathways for spatial navigation in humans: evidence from resting-state functional connectivity. *Brain Struct. Funct.* 222 1945–1957. 10.1007/s00429-016-1318-6 27704218

[B9] Boccia M. Sulpizio V. Palermo L. Piccardi L. Guariglia C. Galati G. (2017b). I can see where you would be: patterns of fMRI activity reveal imagined landmarks. *Neuroimage* 144 174–182. 10.1016/j.neuroimage.2016.08.034 27554528

[B10] Boccia M. Sulpizio V. Teghil A. Palermo L. Piccardi L. Galati G. (2019). The dynamic contribution of the high-level visual cortex to imagery and perception. *Hum. Brain Mapp.* 40 2449–2463. 10.1002/hbm.24535 30702203 PMC6865452

[B11] Bouwmeester L. Van De Wege A. Haaxma R. Snoek J. W. (2015). Rehabilitation in a complex case of topographical disorientation. *Neuropsychol. Rehabil.* 25 1–14. 10.1080/09602011.2014.923318 24885419

[B12] Buzsaki G. Moser E. I. (2013). Memory, navigation and theta rhythm in the hippocampal-entorhinal system. *Nat. Neurosci.* 16 130–138. 10.1038/nn.3304 23354386 PMC4079500

[B13] Byrne P. Becker S. Burgess N. (2007). Remembering the past and imagining the future: a neural model of spatial memory and imagery. *Psychol. Rev.* 114 340–375. 10.1037/0033-295x.114.2.340 17500630 PMC2678675

[B14] Chiaravalloti N. D. Deluca J. Moore N. B. Ricker J. H. (2005). Treating learning impairments improves memory performance in multiple sclerosis: a randomized clinical trial. *Mult. Scler.* 11 58–68. 10.1191/1352458505ms1118oa 15732268

[B15] Conson M. Bianchini F. Quarantelli M. Boccia M. Salzano S. Di Vita A. (2018). Selective map-following navigation deficit: a new case of developmental topographical disorientation. *J. Clin. Exp. Neuropsychol.* 40 940–950. 10.1080/13803395.2018.1451493 29614925

[B16] Corsi P. M. (1972). *Human Memory and the Medial Temporal Region of the Brain.* Ph.D. thesis, McGill University, Montreal, QC.

[B17] Crawford J. R. Garthwaite P. H. (2002). Investigation of the single case in neuropsychology: confidence limits on the abnormality of test scores and test score differences. *Neuropsychologia* 40 1196–1208. 10.1016/s0028-3932(01)00224-x 11931923

[B18] Crawford J. R. Garthwaite P. H. Ryan K. (2011). Comparing a single case to a control sample: testing for neuropsychological deficits and dissociations in the presence of covariates. *Cortex* 47 1166–1178. 10.1016/j.cortex.2011.02.017 21458788

[B19] Davis S. J. C. Coltheart M. (1999). Rehabilitation of topographical disorientation: an experimental single case study. *Neuropsychol. Rehabil.* 9 1–30. 10.1080/713755586

[B20] Ekstrom A. D. Huffman D. J. Starrett M. (2017). Interacting networks of brain regions underlie human spatial navigation: a review and novel synthesis of the literature. *J. Neurophys.* 118 3328–3344. 10.1152/jn.00531.2017 28931613 PMC5814720

[B21] Ekstrom A. D. Kahana M. J. Caplan J. B. Fields T. A. Isham E. A. Newman E. L. (2003). Cellular networks underlying human spatial navigation. *Nature* 425 184–188. 10.1038/nature01964 12968182

[B22] Holmes G. Horrax G. (1919). Disturbances of spatial orientation and visual attention, with loss of stereoscopic vision. *Arch. Neurol. Psychiatr.* 1 385–407.

[B23] Iaria G. Barton J. J. (2010). Developmental topographical disorientation: a newly discovered cognitive disorder. *Exp. Brain Res.* 206 189–196. 10.1007/s00221-010-2256-9 20431873

[B24] Iaria G. Chen J. K. Guariglia C. Ptito A. Petrides M. (2007). Retrosplenial and hippocampal brain regions in human navigation: complementary functional contributions to the formation and use of cognitive maps. *Eur. J. Neurosci.* 25 890–899. 10.1111/j.1460-9568.2007.05371.x 17298595

[B25] Incoccia C. Magnotti L. Iaria G. Piccardi L. Guariglia C. (2009). Topographical disorientation in a patient who never developed navigational skills: the (re)habilitation treatment. *Neuropsychol. Rehabil.* 19 291–314. 10.1080/09602010802188344 18773313

[B26] Kaschel R. Della Sala S. Cantagallo A. Fahlböck A. Laaksonen R. Kazen M. (2002). Imagery mnemonics for the rehabilitation of memory: a randomised group controlled trial. *Neuropsychol. Rehabil.* 12 127–153. 10.1080/09602010143000211

[B27] Kravitz D. J. Saleem K. S. Baker C. I. Mishkin M. (2011). A new neural framework for visuospatial processing. *Nat. Rev. Neurosci.* 12 217–230. 10.1038/nrn3008 21415848 PMC3388718

[B28] Levine D. N. Warach J. Farah M. (1985). Two visual systems in mental imagery: dissociation of “what” and “where” in imagery disorders due to bilateral posterior cerebral lesions. *Neurology* 35 1010–1018. 4010939 10.1212/wnl.35.7.1010

[B29] Money J. Alexander D. Walker H. T. (1965). *A Standardized Road-Map Test of Direction Sense; Manual.* Baltimore: Johns Hopkins Press.

[B30] Montello D. R. (1993). “Scale and multiple psychologies of space in spatial information theory: a theoretical basis for GIS,” in *Proceedings of the COSIT: European Conference on Spatial Information Theory* (Berlin: Springer-Verlag), 312–321. 10.1007/3-540-57207-4_21

[B31] O’Keefe J. Dostrovsky J. (1971). The hippocampus as a spatial map. Preliminary evidence from unit activity in the freely-moving rat. *Brain Res.* 34 171–175. 10.1016/0006-8993(71)90358-1 5124915

[B32] Palermo L. Piccardi L. Bianchini F. Nemmi F. Giorgio V. Incoccia C. (2014). Looking for the compass in a case of developmental topographical disorientation: a behavioral and neuroimaging study. *J. Clin. Exp. Neuropsychol.* 36 464–481. 10.1080/13803395.2014.904843 24742227

[B33] Palermo L. Piccardi L. Nori R. Giusberti F. Guariglia C. (2016). The impact of ageing and gender on visual mental imagery processes: a study of performance on tasks from the complete visual mental imagery battery (CVMIB). *J. Clin. Exp. Neuropsychol.* 38 752–763. 10.1080/13803395.2016.1161735 27134072

[B34] Piccardi L. Bianchini F. Argento O. De Nigris A. Maialetti A. Palermo L. (2013). The Walking Corsi Test (WalCT): standardization of the topographical memory test in an Italian population. *Neurol. Sci.* 34 971–978. 10.1007/s10072-012-1175-x 22903771

[B35] Piccardi L. Iaria G. Ricci M. Bianchini F. Zompanti L. Guariglia C. (2008). Walking in the Corsi test: which type of memory do you need? *Neurosci. Lett.* 432 127–131. 10.1016/j.neulet.2007.12.044 18226450

[B36] Piras F. Borella E. Incoccia C. Carlesimo G. A. (2011). Evidence-based practice recommendations for memory rehabilitation. *Eur. J. Phys. Rehabil. Med.* 47:27. 21448125

[B37] Siegel A. W. White S. H. (1975). *The Development of Spatial Representations of Large-Scale Environments.* Amsterdam: Academic Press. 10.1016/s0065-2407(08)60007-5 1101663

[B38] Stark M. Coslett H. B. Saffran E. M. (1996). Impairment of an egocentric map of locations: implications for perception and action. *Cogn. Neuropsychol.* 13 481–523.

[B39] Sulpizio V. Boccia M. Guariglia C. Galati G. (2017). Implicit coding of location and direction in a familiar, real-world “vista” space. *Behav. Brain Res.* 319 16–24. 10.1016/j.bbr.2016.10.052 27840248

[B40] Sulpizio V. Boccia M. Guariglia C. Galati G. (2018). Neural codes for one’s own position and direction in a real-world “vista” environment. *Front. Hum. Neurosci.* 12:167. 10.3389/fnhum.2018.00167 PMC5936771 29760655

[B41] Sulpizio V. Committeri G. Galati G. (2014). Distributed cognitive maps reflecting real distances between places and views in the human brain. *Front. Hum. Neurosci.* 8:716. 10.3389/fnhum.2014.00716 25309392 PMC4160952

[B42] Svoboda E. Mccarthy J. Moscovitch M. (2018). A case study of topographical disorientation: behavioural intervention for achieving independent navigation AU - Rivest, Josée. *Neuropsychol. Rehabil.* 28 797–817. 10.1080/09602011.2016.1160833 27027908

[B43] Takahashi N. Kawamura M. Shiota J. Kasahata N. Hirayama K. (1997). Pure topographic disorientation due to right retrosplenial lesion. *Neurology* 49 464–469. 10.1212/wnl.49.2.464 9270578

[B44] Taube J. S. (1998). Head direction cells and the neurophysiological basis for a sense of direction. *Prog. Neurobiol.* 55 225–256. 10.1016/s0301-0082(98)00004-5 9643555

[B45] Thurstone L. L. (1937). Ability, motivation, and speed. *Psychometrika* 2 249–254. 10.1007/bf02287896

[B46] Vass L. K. Epstein R. A. (2013). Abstract representations of location and facing direction in the human brain. *J. Neurosci.* 33 6133–6142. 10.1523/JNEUROSCI.3873-12.2013 23554494 PMC3656495

[B47] Wang R. F. Spelke E. S. (2002). Human spatial representation: insights from animals. *Trends Cogn. Sci.* 6 376–382. 10.1016/s1364-6613(02)01961-7 12200179

[B48] Wilson B. A. Greenfield E. Clare L. Baddeley A. Cockburn J. Watson P. (2008). *Rivermead Behavioral Memory Test*, 3rd Edn London: Thames Valley Test Company.

[B49] Wolbers T. Hegarty M. (2010). What determines our navigational abilities? *Trends Cogn. Sci.* 14 138–146. 10.1016/j.tics.2010.01.001 20138795

[B50] Wolbers T. Wiener J. M. (2014). Challenges for identifying the neural mechanisms that support spatial navigation: the impact of spatial scale. *Front. Hum. Neurosci.* 8:571. 10.3389/fnhum.2014.00571 25140139 PMC4121531

