# Supplementary material for: Topographical Disorientation: Clinical and Theoretical Significance of Long-Lasting Improvements Following Imagery-Based Training
Source: Front Hum Neurosci. 2019 Sep 20;13:322. doi: 10.3389/fnhum.2019.00322 (PMC6764239; doi:10.3389/fnhum.2019.00322)
Supplement: Supplementary file 1 [file Data_Sheet_1.PDF]

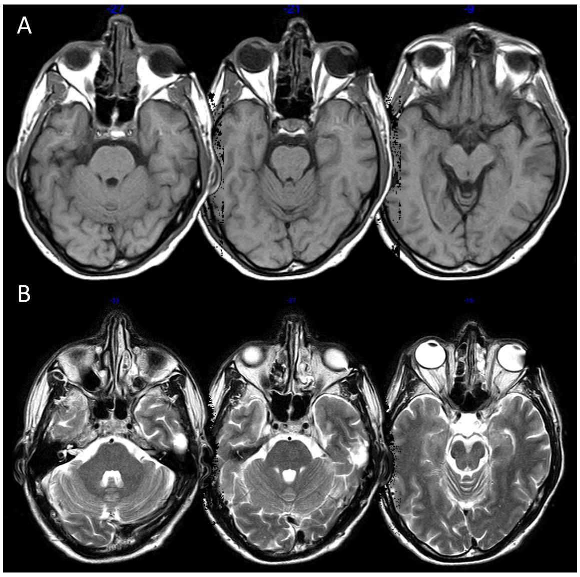

Supplementary Figure 1 T1 and T2-weighted images (panel A and B respectively)

Imaging data

The computerized tomography performed immediately after the event revealed sites of contusion in the right temporal lobe. This finding was confirmed 4 years later by magnetic resonance imaging: T2-weighted images revealed hyperintensities of right temporal lobe extending also to subcortical areas (Supplementary Figure 1)

Phases of the imagery-based treatment (IBT)

First phase: motivation for imagery training and rapid generation of mental images

The first session was aimed at motivating NLL to the IBT and consolidating the ability to quickly generate a Mental Imagery after a verbal input. A number of objects of increasing complexity (e.g. an airplane; a piano; a guitar; a banknote) were chosen and questions about specific characteristic of these objects were asked (e.g. Are there any pedals inside the cockpit? Where are the strings in the piano? Do the banknotes have a signature?).

Second phase: generation and retrieval of navigational knowledge

Since the second session, the abilities consolidated during the first phase were applied to spatial knowledge acquisition, by following the hierarchical stages proposed by Siegel and White (Siegel and White, 1975), namely landmark, route and survey representation. Each level was initially prompted by using well-known familiar points of interest (e.g. churches, monuments, squares); then NLL's personal points of interest (POI) were used (e.g., workplace, preferred restaurant, supermarket).

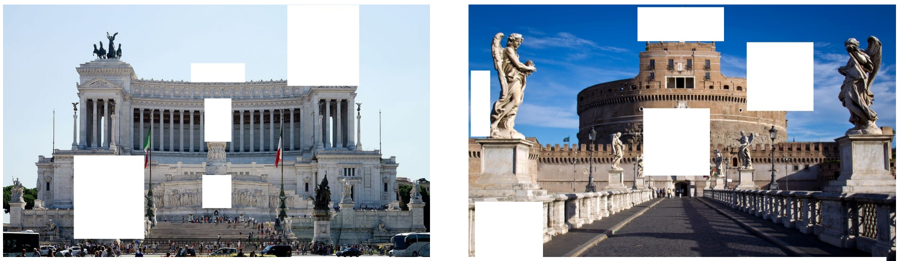

Supplementary Figure 2 Example of stimuli used in the picture completion task used to prompt landmark representation.

Landmark representation. NLL was asked to represent as vividly as possible the mental image of an item (e.g. a monument or a POI) following a verbal input (e.g., Imagine the Colosseum). Thus, specific questions about pictorial features of that item was asked (e.g. What color is the Colosseum? Are there any plates or engravings? Where is the entrance?). This task prompted the inspection of the topological mental image. Then, according to the view-dependent recognition system proposed by Wang & Spelke (2002), we asked NLL specific questions tapping on specific viewpoints of the monument/POI (i.e. Coming from the Arch of Constantine, what part of the Colosseum do you see in front?) and the surrounding spatial context

(e.g. Are there any green areas nearby? Where is the metro station located?). Landmark representation was also prompted by a picture completion task: images representing familiar landmarks were modified, applying a white patch to mask specific part of the image (missing object). After observing the modified image, NLL had to construct the mental image of the landmark and to report the missing object (Supplementary Figure 2).

Route representation. NLL was asked to imagine and describe a path, guided by specific indications, trying to focus on the salient points that he met along the path. The first questions focused on directional information (e.g. What is on the right side watching at St. Peter's cathedral? And on the left?); then, we asked NLL to retrieve specific routes (e.g., how can I get to the Supreme Court from the Mole Adriana?). Google Street View was used to deal with initial difficulties in this task: NLL was provided with a piece of the path and asked to continue once it was stopped.

Survey representation. Finally, we focused on the egocentric perspective taking and the acquisition of the environmental survey representation: NLL was asked to imagine himself outside the Santa Lucia foundation, targeting specific environmental objects (e.g. Where is the Appia Antica? Where is the Circus Maximus?). He was also asked about the distances between monuments/POIs (e.g. is Fontana di Trevi nearest to Piazza Venezia or San Pietro?). Finally, NLL was asked to draw on a map the paths between different monuments/POIs.
